# 1,5-Dibenzyl-3-propargyl-1,5-benzo­diazepine-2,4-dione

**DOI:** 10.1107/S1600536809048508

**Published:** 2009-11-21

**Authors:** Hind Jabli, F. Ouazzani Chahdi, Bernard Garrigues, El Mokhtar Essassi, Seik Weng Ng

**Affiliations:** a Laboratoire de Chimie Organique Appliquée, Faculté des Sciences et Techniques, Université Sidi Mohamed Ben Abdallah, Fés, Morocco; b Laboratoire de Hétérochimie Fondamentale et Appliquée, Université Paul Sabatier, Toulouse, France; c Laboratoire de Chimie Organique Hétérocyclique, Pôle de compétences Pharmacochimie, Université Mohammed V-Agdal, B.P. 1014 Avenue Ibn Batout, Rabat, Morocco; d Department of Chemistry, University of Malaya, 50603 Kuala Lumpur, Malaysia

## Abstract

The title compound, C_26_H_22_N_2_O_2_, features a benzene ring fused with a seven-membered diazepine ring; the latter ring adopts a boat conformation (with the propargylallyl-bearing C atom as the prow and the fused-ring C atoms as the stern). The phenyl ring of one of the two benzyl substituents is disordered over two positions in a 0.812 (11):0.188 (11) ratio.

## Related literature

For the crystal structure of the parent compound, benzodiazepin-2,4-dione, see: Négrier *et al.* (2006 ▶).
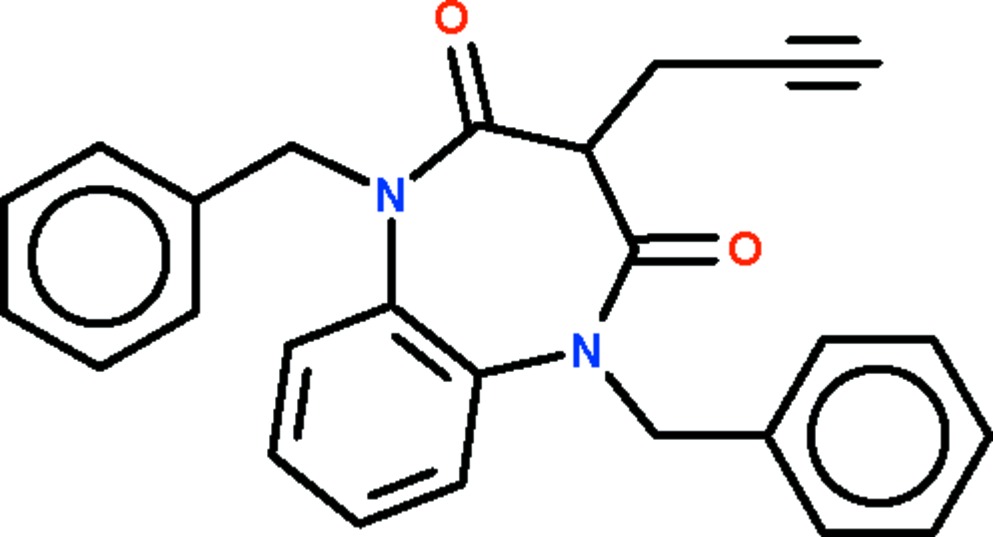


## Experimental

### 

#### Crystal data

C_26_H_22_N_2_O_2_
*M* *_r_* = 394.46
Monoclinic, 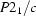

*a* = 8.8663 (2) Å
*b* = 18.6771 (4) Å
*c* = 12.4665 (3) Å
β = 91.154 (1)°
*V* = 2063.99 (8) Å^3^
*Z* = 4
Mo *K*α radiation
μ = 0.08 mm^−1^
*T* = 193 K
0.70 × 0.50 × 0.30 mm

#### Data collection

Bruker APEXII diffractometer
Absorption correction: none
29296 measured reflections
4750 independent reflections
3931 reflections with *I* > 2σ(*I*)
*R*_int_ = 0.027

#### Refinement

*R*[*F* ^2^ > 2σ(*F* ^2^)] = 0.040
*wR*(*F* ^2^) = 0.112
*S* = 1.02
4750 reflections
278 parameters
H-atom parameters constrained
Δρ_max_ = 0.26 e Å^−3^
Δρ_min_ = −0.27 e Å^−3^

### 

Data collection: *APEX2* (Bruker, 2005 ▶); cell refinement: *SAINT* (Bruker, 2005 ▶); data reduction: *SAINT*; program(s) used to solve structure: *SHELXS97* (Sheldrick, 2008 ▶); program(s) used to refine structure: *SHELXL97* (Sheldrick, 2008 ▶); molecular graphics: *X-SEED* (Barbour, 2001 ▶); software used to prepare material for publication: *publCIF* (Westrip, 2009 ▶).

## Supplementary Material

Crystal structure: contains datablocks global, I. DOI: 10.1107/S1600536809048508/sj2679sup1.cif

Structure factors: contains datablocks I. DOI: 10.1107/S1600536809048508/sj2679Isup2.hkl

Additional supplementary materials:  crystallographic information; 3D view; checkCIF report

## Figures and Tables

**Fig. 1.**
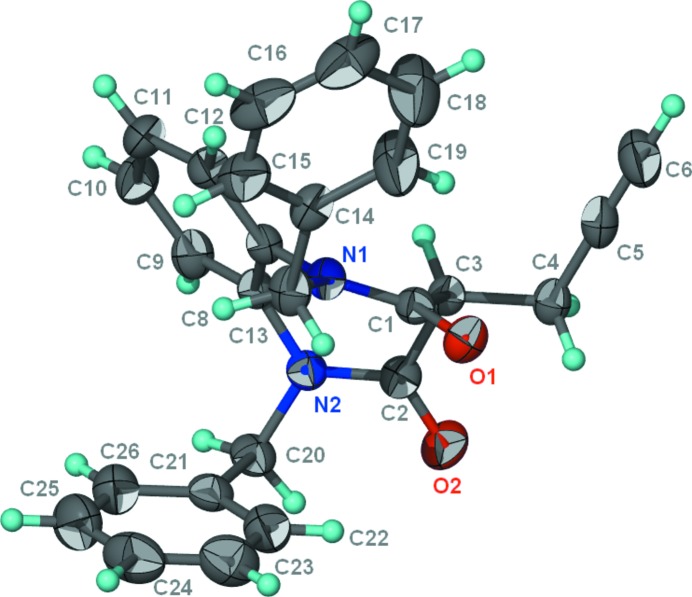
Thermal ellipsoid plot (Barbour, 2001) of C26H22N2O2 at the 70% probability level; hydrogen atoms are drawn as spheres of arbitrary radius. The disorder in one of the phenyl rings is not shown.

## supplementary crystallographic information

### Experimental

To a solution of the potassium *t*-butoxide (0.24 g, 2.13 mmol) in THF (15 ml) was added 1,5-dibenzyl-1,5-benzodiazepine-2,4-dione (0.50 g, 1.40 mmol) and propargyl bromide (0.20 ml, 1.88 mmol). Stirring was continued for 24 h. The reaction was monitored by thin layer chromatography. The mixture was filtered and the solution evaporated to give colorless crystals.

A somewhat large crystal was used in the diffraction measurements.

### Refinement

Carbon-bound H-atoms were placed in calculated positions (C—H 0.95 to 1.00 Å) and were included in the refinement in the riding model approximation, with *U*(H) set to 1.2*U*(C).

One of the phenyl rings is disordered over two positions. This was refined as two rigid hexagons of 1.39 Å sides. The temperature factors of the primed atoms were restrained to those of the unprimed ones. The minor component refined to 0.188 (11).

### Crystal data

**Table:**

C_26_H_22_N_2_O_2_	*F*(000) = 832
*M**_r_* = 394.46	*D*_x_ = 1.269 Mg m^−^^3^
Monoclinic, *P*2_1_/*c*	Mo *K*α radiation, λ = 0.71073 Å
Hall symbol: -P 2ybc	Cell parameters from 9973 reflections
*a* = 8.8663 (2) Å	θ = 2.5–32.9°
*b* = 18.6771 (4) Å	µ = 0.08 mm^−^^1^
*c* = 12.4665 (3) Å	*T* = 193 K
β = 91.154 (1)°	Irregular block, colorless
*V* = 2063.99 (8) Å^3^	0.70 × 0.50 × 0.30 mm
*Z* = 4	

### Data collection

**Table:**

Bruker APEXII diffractometer	3931 reflections with *I* > 2σ(*I*)
Radiation source: fine-focus sealed tube	*R*_int_ = 0.027
graphite	θ_max_ = 27.5°, θ_min_ = 2.0°
φ and ω scans	*h* = −11→11
29296 measured reflections	*k* = −24→18
4750 independent reflections	*l* = −16→15

### Refinement

**Table:**

Refinement on *F*^2^	Primary atom site location: structure-invariant direct methods
Least-squares matrix: full	Secondary atom site location: difference Fourier map
*R*[*F*^2^ > 2σ(*F*^2^)] = 0.040	Hydrogen site location: inferred from neighbouring sites
*wR*(*F*^2^) = 0.112	H-atom parameters constrained
*S* = 1.02	*w* = 1/[σ^2^(*F*_o_^2^) + (0.0579*P*)^2^ + 0.5557*P*] where *P* = (*F*_o_^2^ + 2*F*_c_^2^)/3
4750 reflections	(Δ/σ)_max_ = 0.001
278 parameters	Δρ_max_ = 0.26 e Å^−^^3^
0 restraints	Δρ_min_ = −0.27 e Å^−^^3^

### Fractional atomic coordinates and isotropic or equivalent isotropic displacement parameters (Å^2^)

**Table:**

	*x*	*y*	*z*	*U*_iso_*/*U*_eq_	Occ. (<1)
O1	0.17022 (9)	0.29934 (4)	0.63891 (7)	0.0310 (2)	
O2	−0.09780 (10)	0.44893 (5)	0.68067 (8)	0.0408 (2)	
N1	0.34007 (10)	0.38740 (5)	0.67524 (7)	0.0250 (2)	
N2	0.12855 (11)	0.50134 (5)	0.71936 (7)	0.0264 (2)	
C1	0.20965 (12)	0.36186 (6)	0.62971 (8)	0.0238 (2)	
C2	0.03474 (13)	0.45816 (6)	0.66023 (9)	0.0268 (2)	
C3	0.11097 (12)	0.41712 (6)	0.57026 (8)	0.0238 (2)	
H3	0.1753	0.4501	0.5277	0.029*	
C4	−0.00697 (13)	0.38106 (6)	0.49721 (9)	0.0285 (2)	
H4A	−0.0646	0.3461	0.5398	0.034*	
H4B	−0.0787	0.4178	0.4701	0.034*	
C5	0.05908 (14)	0.34408 (7)	0.40583 (10)	0.0334 (3)	
C6	0.11070 (17)	0.31415 (8)	0.33138 (12)	0.0461 (3)	
H6	0.1521	0.2901	0.2716	0.055*	
C7	0.38435 (13)	0.46069 (6)	0.67074 (8)	0.0249 (2)	
C8	0.28132 (13)	0.51554 (6)	0.69185 (8)	0.0255 (2)	
C9	0.33036 (15)	0.58677 (7)	0.68953 (10)	0.0332 (3)	
H9	0.2605	0.6244	0.7011	0.040*	
C10	0.47975 (16)	0.60272 (7)	0.67056 (10)	0.0384 (3)	
H10	0.5122	0.6512	0.6700	0.046*	
C11	0.58233 (15)	0.54818 (7)	0.65236 (10)	0.0369 (3)	
H11	0.6853	0.5593	0.6410	0.044*	
C12	0.53473 (14)	0.47752 (7)	0.65072 (9)	0.0314 (3)	
H12	0.6046	0.4404	0.6359	0.038*	
C13	0.43658 (14)	0.33760 (7)	0.73776 (10)	0.0320 (3)	
H13A	0.4796	0.3649	0.7991	0.038*	0.188 (11)
H13B	0.3695	0.3008	0.7681	0.038*	0.188 (11)
H13C	0.3715	0.3028	0.7748	0.038*	0.812 (11)
H13D	0.4930	0.3650	0.7934	0.038*	0.812 (11)
C14'	0.5525 (9)	0.3026 (8)	0.6915 (10)	0.0290 (5)	0.188 (11)
C15'	0.6972 (11)	0.3031 (7)	0.7365 (9)	0.0426 (6)	0.188 (11)
H15'	0.7173	0.3290	0.8008	0.051*	0.188 (11)
C16'	0.8126 (8)	0.2659 (7)	0.6873 (11)	0.0480 (10)	0.188 (11)
H16'	0.9115	0.2663	0.7180	0.058*	0.188 (11)
C17'	0.7832 (12)	0.2281 (6)	0.5931 (12)	0.0511 (8)	0.188 (11)
H17'	0.8621	0.2026	0.5595	0.061*	0.188 (11)
C18'	0.6385 (14)	0.2275 (8)	0.5482 (12)	0.0728 (9)	0.188 (11)
H18'	0.6184	0.2017	0.4838	0.087*	0.188 (11)
C19'	0.5231 (10)	0.2648 (9)	0.5974 (11)	0.0567 (7)	0.188 (11)
H19'	0.4242	0.2644	0.5667	0.068*	0.188 (11)
C14	0.5505 (2)	0.29604 (17)	0.6685 (3)	0.0290 (5)	0.812 (11)
C15	0.6962 (2)	0.28451 (19)	0.7075 (3)	0.0426 (6)	0.812 (11)
H15	0.7261	0.3039	0.7751	0.051*	0.812 (11)
C16	0.7990 (2)	0.2450 (2)	0.6492 (4)	0.0480 (10)	0.812 (11)
H16	0.8979	0.2373	0.6775	0.058*	0.812 (11)
C17	0.7587 (3)	0.21739 (14)	0.5519 (4)	0.0511 (8)	0.812 (11)
H17	0.8288	0.1903	0.5121	0.061*	0.812 (11)
C18	0.6154 (3)	0.22919 (19)	0.5119 (4)	0.0728 (9)	0.812 (11)
H18	0.5870	0.2105	0.4436	0.087*	0.812 (11)
C19	0.5119 (3)	0.2679 (2)	0.5699 (3)	0.0567 (7)	0.812 (11)
H19	0.4131	0.2752	0.5412	0.068*	0.812 (11)
C20	0.07850 (14)	0.52656 (7)	0.82584 (9)	0.0313 (3)	
H20A	−0.0327	0.5235	0.8295	0.038*	
H20B	0.1083	0.5772	0.8365	0.038*	
C21	0.15023 (14)	0.48060 (7)	0.91273 (9)	0.0305 (3)	
C22	0.11027 (17)	0.40885 (7)	0.92212 (10)	0.0396 (3)	
H22	0.0354	0.3893	0.8751	0.047*	
C23	0.17857 (19)	0.36561 (8)	0.99950 (12)	0.0482 (4)	
H23	0.1511	0.3166	1.0048	0.058*	
C24	0.28666 (18)	0.39393 (9)	1.06888 (11)	0.0505 (4)	
H24	0.3327	0.3645	1.1224	0.061*	
C25	0.32761 (19)	0.46476 (10)	1.06047 (12)	0.0522 (4)	
H25	0.4021	0.4841	1.1080	0.063*	
C26	0.25989 (16)	0.50798 (8)	0.98237 (11)	0.0415 (3)	
H26	0.2890	0.5568	0.9767	0.050*	

### Atomic displacement parameters (Å^2^)

**Table:**

	*U*^11^	*U*^22^	*U*^33^	*U*^12^	*U*^13^	*U*^23^
O1	0.0302 (4)	0.0243 (4)	0.0384 (4)	−0.0013 (3)	−0.0020 (3)	0.0049 (3)
O2	0.0226 (4)	0.0483 (6)	0.0516 (6)	0.0013 (4)	0.0039 (4)	−0.0121 (4)
N1	0.0235 (5)	0.0243 (5)	0.0272 (4)	0.0033 (4)	−0.0028 (4)	0.0014 (3)
N2	0.0249 (5)	0.0275 (5)	0.0269 (5)	0.0028 (4)	0.0014 (4)	−0.0024 (4)
C1	0.0232 (5)	0.0249 (5)	0.0233 (5)	0.0015 (4)	0.0013 (4)	0.0008 (4)
C2	0.0236 (6)	0.0262 (5)	0.0306 (5)	0.0040 (4)	−0.0011 (4)	0.0010 (4)
C3	0.0239 (5)	0.0228 (5)	0.0246 (5)	0.0006 (4)	−0.0020 (4)	0.0015 (4)
C4	0.0272 (6)	0.0279 (6)	0.0302 (5)	−0.0007 (4)	−0.0063 (4)	0.0013 (4)
C5	0.0338 (6)	0.0305 (6)	0.0355 (6)	−0.0041 (5)	−0.0096 (5)	−0.0020 (5)
C6	0.0457 (8)	0.0493 (8)	0.0430 (7)	−0.0003 (7)	−0.0068 (6)	−0.0156 (6)
C7	0.0257 (5)	0.0264 (5)	0.0225 (5)	−0.0006 (4)	−0.0021 (4)	−0.0012 (4)
C8	0.0251 (6)	0.0279 (5)	0.0234 (5)	−0.0006 (4)	−0.0018 (4)	−0.0010 (4)
C9	0.0369 (7)	0.0268 (6)	0.0358 (6)	0.0002 (5)	−0.0030 (5)	−0.0029 (5)
C10	0.0424 (7)	0.0309 (6)	0.0417 (7)	−0.0111 (5)	−0.0010 (6)	−0.0012 (5)
C11	0.0301 (6)	0.0435 (7)	0.0371 (6)	−0.0114 (5)	0.0023 (5)	−0.0031 (5)
C12	0.0260 (6)	0.0363 (6)	0.0318 (6)	−0.0005 (5)	0.0013 (4)	−0.0035 (5)
C13	0.0311 (6)	0.0324 (6)	0.0322 (6)	0.0037 (5)	−0.0096 (5)	0.0045 (5)
C14'	0.0251 (6)	0.0232 (8)	0.0384 (13)	0.0018 (5)	−0.0039 (7)	0.0042 (8)
C15'	0.0285 (7)	0.0577 (15)	0.0414 (14)	0.0039 (9)	−0.0049 (8)	0.0094 (11)
C16'	0.0246 (8)	0.0538 (16)	0.066 (2)	0.0098 (9)	0.0024 (10)	0.0223 (16)
C17'	0.0350 (11)	0.0326 (9)	0.086 (2)	0.0053 (8)	0.0170 (12)	−0.0031 (11)
C18'	0.0433 (12)	0.0895 (15)	0.086 (2)	0.0116 (11)	−0.0022 (13)	−0.0534 (17)
C19'	0.0300 (8)	0.0751 (12)	0.0647 (18)	0.0138 (8)	−0.0097 (9)	−0.0338 (14)
C14	0.0251 (6)	0.0232 (8)	0.0384 (13)	0.0018 (5)	−0.0039 (7)	0.0042 (8)
C15	0.0285 (7)	0.0577 (15)	0.0414 (14)	0.0039 (9)	−0.0049 (8)	0.0094 (11)
C16	0.0246 (8)	0.0538 (16)	0.066 (2)	0.0098 (9)	0.0024 (10)	0.0223 (16)
C17	0.0350 (11)	0.0326 (9)	0.086 (2)	0.0053 (8)	0.0170 (12)	−0.0031 (11)
C18	0.0433 (12)	0.0895 (15)	0.086 (2)	0.0116 (11)	−0.0022 (13)	−0.0534 (17)
C19	0.0300 (8)	0.0751 (12)	0.0647 (18)	0.0138 (8)	−0.0097 (9)	−0.0338 (14)
C20	0.0313 (6)	0.0314 (6)	0.0313 (6)	0.0061 (5)	0.0046 (5)	−0.0057 (5)
C21	0.0294 (6)	0.0350 (6)	0.0273 (5)	0.0027 (5)	0.0070 (4)	−0.0035 (4)
C22	0.0442 (8)	0.0391 (7)	0.0355 (6)	−0.0043 (6)	0.0008 (5)	−0.0016 (5)
C23	0.0620 (10)	0.0395 (7)	0.0433 (7)	0.0013 (7)	0.0074 (7)	0.0067 (6)
C24	0.0524 (9)	0.0622 (10)	0.0369 (7)	0.0101 (8)	0.0009 (6)	0.0118 (7)
C25	0.0473 (9)	0.0694 (10)	0.0394 (7)	−0.0062 (8)	−0.0092 (6)	0.0018 (7)
C26	0.0430 (8)	0.0436 (7)	0.0378 (7)	−0.0062 (6)	−0.0010 (6)	−0.0024 (6)

### Geometric parameters (Å, °)

**Table:**

O1—C1	1.2248 (13)	C15'—C16'	1.3900
O2—C2	1.2197 (15)	C15'—H15'	0.9500
N1—C1	1.3640 (14)	C16'—C17'	1.3900
N1—C7	1.4254 (14)	C16'—H16'	0.9500
N1—C13	1.4758 (14)	C17'—C18'	1.3900
N2—C2	1.3641 (15)	C17'—H17'	0.9500
N2—C8	1.4288 (15)	C18'—C19'	1.3900
N2—C20	1.4847 (14)	C18'—H18'	0.9500
C1—C3	1.5342 (14)	C19'—H19'	0.9500
C2—C3	1.5274 (15)	C14—C19	1.373 (2)
C3—C4	1.5291 (15)	C14—C15	1.388 (2)
C3—H3	1.0000	C15—C16	1.389 (3)
C4—C5	1.4645 (18)	C15—H15	0.9500
C4—H4A	0.9900	C16—C17	1.359 (3)
C4—H4B	0.9900	C16—H16	0.9500
C5—C6	1.1833 (19)	C17—C18	1.373 (3)
C6—H6	0.9500	C17—H17	0.9500
C7—C12	1.3972 (17)	C18—C19	1.384 (3)
C7—C8	1.4010 (16)	C18—H18	0.9500
C8—C9	1.4001 (17)	C19—H19	0.9500
C9—C10	1.3826 (19)	C20—C21	1.5122 (17)
C9—H9	0.9500	C20—H20A	0.9900
C10—C11	1.387 (2)	C20—H20B	0.9900
C10—H10	0.9500	C21—C26	1.3880 (18)
C11—C12	1.3856 (18)	C21—C22	1.3918 (18)
C11—H11	0.9500	C22—C23	1.388 (2)
C12—H12	0.9500	C22—H22	0.9500
C13—C14'	1.356 (8)	C23—C24	1.383 (2)
C13—C14	1.550 (3)	C23—H23	0.9500
C13—H13A	0.9900	C24—C25	1.376 (2)
C13—H13B	0.9900	C24—H24	0.9500
C13—H13C	0.9900	C25—C26	1.392 (2)
C13—H13D	0.9900	C25—H25	0.9500
C14'—C15'	1.3900	C26—H26	0.9500
C14'—C19'	1.3900		
			
C1—N1—C7	123.46 (9)	C13—C14'—C15'	121.8 (7)
C1—N1—C13	118.49 (9)	C13—C14'—C19'	118.2 (7)
C7—N1—C13	117.96 (9)	C15'—C14'—C19'	120.0
C2—N2—C8	123.41 (9)	C14'—C15'—C16'	120.0
C2—N2—C20	118.73 (10)	C14'—C15'—H15'	120.0
C8—N2—C20	117.23 (9)	C16'—C15'—H15'	120.0
O1—C1—N1	122.39 (10)	C15'—C16'—C17'	120.0
O1—C1—C3	121.70 (10)	C15'—C16'—H16'	120.0
N1—C1—C3	115.86 (9)	C17'—C16'—H16'	120.0
O2—C2—N2	123.38 (11)	C18'—C17'—C16'	120.0
O2—C2—C3	121.66 (10)	C18'—C17'—H17'	120.0
N2—C2—C3	114.81 (10)	C16'—C17'—H17'	120.0
C2—C3—C4	110.55 (9)	C19'—C18'—C17'	120.0
C2—C3—C1	103.86 (8)	C19'—C18'—H18'	120.0
C4—C3—C1	111.59 (9)	C17'—C18'—H18'	120.0
C2—C3—H3	110.2	C18'—C19'—C14'	120.0
C4—C3—H3	110.2	C18'—C19'—H19'	120.0
C1—C3—H3	110.2	C14'—C19'—H19'	120.0
C5—C4—C3	113.06 (10)	C19—C14—C15	117.86 (16)
C5—C4—H4A	109.0	C19—C14—C13	122.45 (17)
C3—C4—H4A	109.0	C15—C14—C13	119.66 (16)
C5—C4—H4B	109.0	C14—C15—C16	120.98 (18)
C3—C4—H4B	109.0	C14—C15—H15	119.5
H4A—C4—H4B	107.8	C16—C15—H15	119.5
C6—C5—C4	179.17 (14)	C17—C16—C15	120.36 (17)
C5—C6—H6	180.0	C17—C16—H16	119.8
C12—C7—C8	119.81 (10)	C15—C16—H16	119.8
C12—C7—N1	119.19 (10)	C16—C17—C18	119.18 (18)
C8—C7—N1	120.91 (10)	C16—C17—H17	120.4
C9—C8—C7	119.14 (11)	C18—C17—H17	120.4
C9—C8—N2	118.53 (10)	C17—C18—C19	120.8 (2)
C7—C8—N2	122.29 (10)	C17—C18—H18	119.6
C10—C9—C8	120.48 (12)	C19—C18—H18	119.6
C10—C9—H9	119.8	C14—C19—C18	120.79 (18)
C8—C9—H9	119.8	C14—C19—H19	119.6
C9—C10—C11	120.21 (12)	C18—C19—H19	119.6
C9—C10—H10	119.9	N2—C20—C21	109.35 (9)
C11—C10—H10	119.9	N2—C20—H20A	109.8
C12—C11—C10	120.08 (12)	C21—C20—H20A	109.8
C12—C11—H11	120.0	N2—C20—H20B	109.8
C10—C11—H11	120.0	C21—C20—H20B	109.8
C11—C12—C7	120.21 (12)	H20A—C20—H20B	108.3
C11—C12—H12	119.9	C26—C21—C22	118.58 (12)
C7—C12—H12	119.9	C26—C21—C20	121.21 (12)
C14'—C13—N1	121.1 (6)	C22—C21—C20	120.18 (11)
N1—C13—C14	113.51 (14)	C23—C22—C21	120.70 (13)
C14'—C13—H13A	107.1	C23—C22—H22	119.7
N1—C13—H13A	107.1	C21—C22—H22	119.7
C14—C13—H13A	116.3	C24—C23—C22	119.96 (14)
C14'—C13—H13B	107.1	C24—C23—H23	120.0
N1—C13—H13B	107.1	C22—C23—H23	120.0
C14—C13—H13B	105.6	C25—C24—C23	120.03 (14)
H13A—C13—H13B	106.8	C25—C24—H24	120.0
C14'—C13—H13C	109.6	C23—C24—H24	120.0
N1—C13—H13C	108.9	C24—C25—C26	120.00 (14)
C14—C13—H13C	108.9	C24—C25—H25	120.0
H13A—C13—H13C	101.4	C26—C25—H25	120.0
N1—C13—H13D	108.9	C21—C26—C25	120.73 (14)
C14—C13—H13D	108.9	C21—C26—H26	119.6
H13C—C13—H13D	107.7	C25—C26—H26	119.6
			
C7—N1—C1—O1	−176.41 (10)	C1—N1—C13—C14	87.11 (17)
C13—N1—C1—O1	−0.01 (16)	C7—N1—C13—C14	−96.30 (16)
C7—N1—C1—C3	0.87 (15)	N1—C13—C14'—C15'	128.9 (7)
C13—N1—C1—C3	177.26 (9)	C14—C13—C14'—C15'	169 (5)
C8—N2—C2—O2	−176.15 (11)	N1—C13—C14'—C19'	−51.8 (9)
C20—N2—C2—O2	13.17 (17)	C14—C13—C14'—C19'	−11 (4)
C8—N2—C2—C3	8.29 (15)	C13—C14'—C15'—C16'	179.3 (12)
C20—N2—C2—C3	−162.38 (9)	C19'—C14'—C15'—C16'	0.0
O2—C2—C3—C4	14.32 (15)	C14'—C15'—C16'—C17'	0.0
N2—C2—C3—C4	−170.04 (9)	C15'—C16'—C17'—C18'	0.0
O2—C2—C3—C1	−105.49 (12)	C16'—C17'—C18'—C19'	0.0
N2—C2—C3—C1	70.14 (11)	C17'—C18'—C19'—C14'	0.0
O1—C1—C3—C2	100.64 (12)	C13—C14'—C19'—C18'	−179.3 (12)
N1—C1—C3—C2	−76.66 (11)	C15'—C14'—C19'—C18'	0.0
O1—C1—C3—C4	−18.46 (15)	C14'—C13—C14—C19	175 (5)
N1—C1—C3—C4	164.24 (9)	N1—C13—C14—C19	−41.8 (3)
C2—C3—C4—C5	176.83 (9)	C14'—C13—C14—C15	−3(4)
C1—C3—C4—C5	−68.12 (12)	N1—C13—C14—C15	140.1 (2)
C1—N1—C7—C12	−137.80 (11)	C19—C14—C15—C16	−0.8 (3)
C13—N1—C7—C12	45.79 (14)	C13—C14—C15—C16	177.4 (2)
C1—N1—C7—C8	45.72 (15)	C14—C15—C16—C17	0.6 (3)
C13—N1—C7—C8	−130.69 (11)	C15—C16—C17—C18	0.2 (3)
C12—C7—C8—C9	1.77 (16)	C16—C17—C18—C19	−0.8 (4)
N1—C7—C8—C9	178.23 (10)	C15—C14—C19—C18	0.2 (3)
C12—C7—C8—N2	−176.05 (10)	C13—C14—C19—C18	−177.9 (3)
N1—C7—C8—N2	0.41 (15)	C17—C18—C19—C14	0.6 (4)
C2—N2—C8—C9	130.25 (12)	C2—N2—C20—C21	99.41 (12)
C20—N2—C8—C9	−58.95 (14)	C8—N2—C20—C21	−71.84 (13)
C2—N2—C8—C7	−51.91 (15)	N2—C20—C21—C26	110.77 (13)
C20—N2—C8—C7	118.89 (11)	N2—C20—C21—C22	−67.23 (15)
C7—C8—C9—C10	−2.35 (17)	C26—C21—C22—C23	0.0 (2)
N2—C8—C9—C10	175.55 (11)	C20—C21—C22—C23	178.08 (12)
C8—C9—C10—C11	0.75 (19)	C21—C22—C23—C24	0.6 (2)
C9—C10—C11—C12	1.46 (19)	C22—C23—C24—C25	−0.7 (2)
C10—C11—C12—C7	−2.03 (19)	C23—C24—C25—C26	0.2 (2)
C8—C7—C12—C11	0.40 (17)	C22—C21—C26—C25	−0.5 (2)
N1—C7—C12—C11	−176.12 (10)	C20—C21—C26—C25	−178.52 (13)
C1—N1—C13—C14'	93.9 (7)	C24—C25—C26—C21	0.4 (2)
C7—N1—C13—C14'	−89.5 (7)		
